# Designing Multistimuli-Responsive Anisotropic Bilayer Hydrogel Actuators by Integrating LCST Phase Transition and Photochromic Isomerization

**DOI:** 10.3390/polym15030786

**Published:** 2023-02-03

**Authors:** Shijun Long, Jiacheng Huang, Jiaqiang Xiong, Chang Liu, Fan Chen, Jie Shen, Yiwan Huang, Xuefeng Li

**Affiliations:** 1 Hubei Provincial Key Laboratory of Green Materials for Light Industry, Hubei University of Technology, Wuhan 430068, China; 2 New Materials and Green Manufacturing Talent Introduction and Innovation Demonstration Base, Hubei University of Technology, Wuhan 430068, China; 3 Hubei Research and Design Institute of Chemical Industry, Wuhan 430073, China

**Keywords:** anisotropic structure, photochromic hydrogels, poly(*N*-isopropylacrylamide), stimuli-responsive hydrogels, soft actuators

## Abstract

Stimuli-responsive hydrogel actuators have attracted tremendous interest in switches and microrobots. Based on *N*-isopropylacrylamide (NIPAM) monomers with LCST phase separation and photochromic molecule spiropyran which can respond to ultraviolet light and H+, we develop a novel multistimuli-responsive co-polymer anisotropic bilayer hydrogel, which can undergo complex deformation behavior under environmental stimuli. Diverse bending angles were achieved based on inhomogeneous swelling. By controlling the environmental temperature, the bilayer hydrogels achieved bending angles of 83.4° and −162.4° below and above the critical temperature of PNIPAM. Stimulated by ultraviolet light and H+, the bilayer hydrogels showed bending angles of −19.4° and −17.3°, respectively. In addition, we designed a strategy to enhance the mechanical properties of the hydrogel via double network (DN). The mechanical properties and microscopic Fourier transform infrared (micro-FTIR) spectrum showed that the bilayer hydrogel can be well bonded at the interfaces of such bilayers. This work will inspire the design and fabrication of novel soft actuators with synergistic functions.

## 1. Introduction

Stimuli-responsive hydrogels can respond to external stimuli through changes in their structures or properties. External stimuli include environmental temperature [1,2,3], light [4,5,6], electricity [7,8], magnetism [9,10], pH [11,12], and chemicals [13]. Stimuli-responsive hydrogels have attracted great attention and shown promising applications in many fields, such as information recording [14], sensors [2,15,16], actuators [2,3,4,5], and biomedicine [12,16,17,18]. The driving force of the hydrogel actuator is mainly derived from swelling/shrinking deformation. It is well known that two main approaches have been developed to achieve complex deformation, uneven stimulation and anisotropic structures. Specifically, the design of anisotropic structure is the basis of the development of hydrogel actuator [19,20]. In recent years, bilayer hydrogel actuators with different swelling rates have been widely studied [21,22,23,24]. Due to their asymmetric response characteristics, the shape deformation can be obtained [25,26,27]. Bilayer hydrogels can be obtained by layer-by-layer polymerization or macroscopic assembly by host-guest interactions or hydrogen bonds as reversible switches. However, the currently investigated bilayer structure can only show simple swelling/shrinking deformation under unitary external stimuli, as it is challenging to integrate complex shape deformation properties in one system. 

**Scheme 1:**
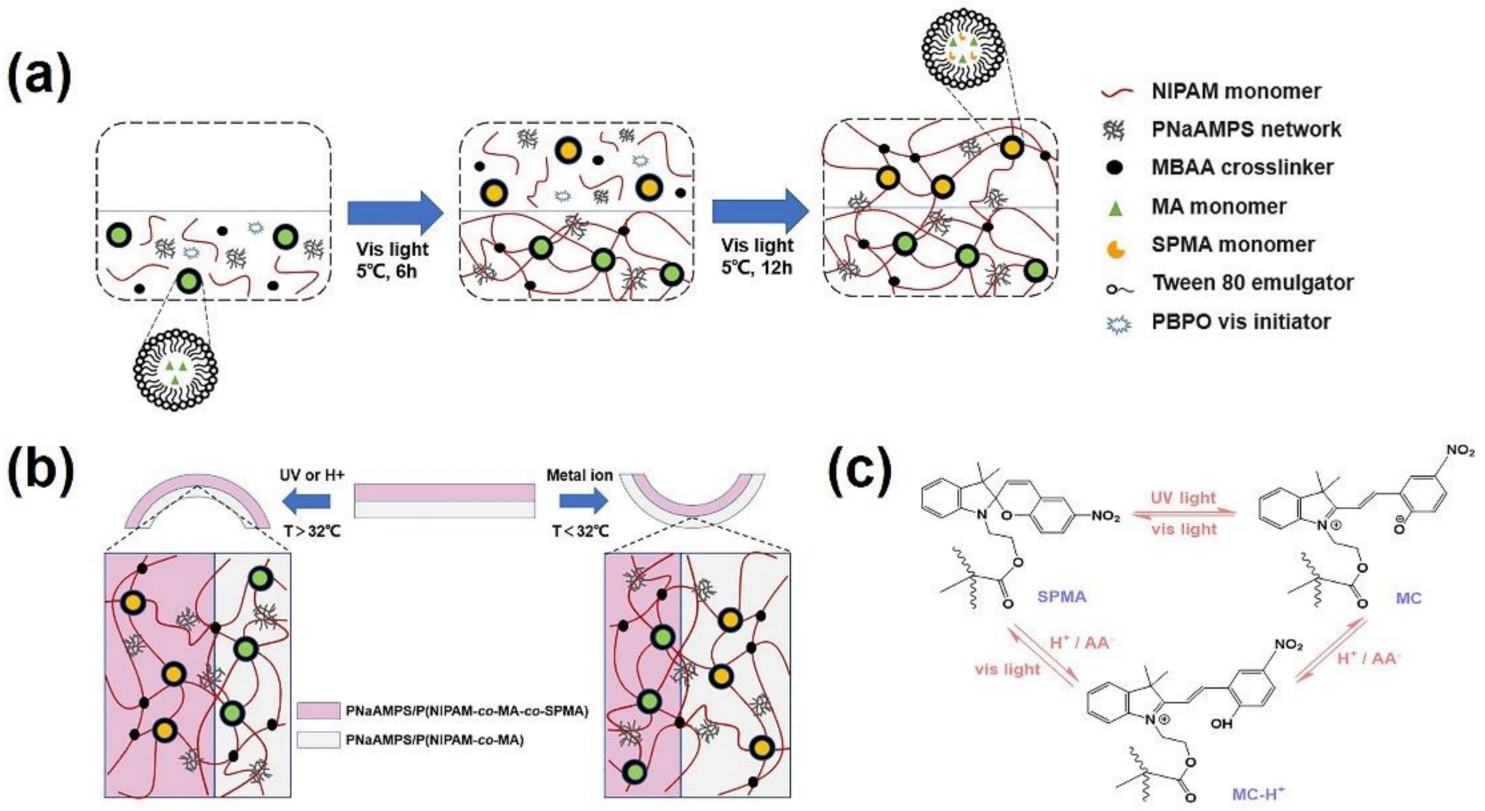
(**a**) Schematic illustration of multistimuli-responsive high-strength bilayer hydrogel (Green represents PNaAMPS/P(NIPAM-*co*-MA) molecule, and orange represents PNaAMPS/P(NIPAM-*co*-MA-*co*-SPMA) molecule); (**b**) multistimuli-responsive mechanisms of bilayer hydrogel; (**c**) photochromism and acidochromism of spiropyran.

In order to realize the utility and functionality of polymeric hydrogel actuators, it is advantageous to develop bilayer hydrogels which can respond to multiple stimuli. Herein, we present a strategy to design and synthesize bilayer hydrogel actuators that comprise thermo-responsive poly(*N*-isopropylacrylamide) (PNIPAM) and UV-light or acid-responsive monomeric spiropyran (SPMA) composition. The actuators are rendered responsive to multiple stimuli by selecting appropriate functional polymers and integrating them into the actuators. PNIPAM has an LCST-type volume phase transition, when the temperature is lower than LCST, the polymer chain of PNIPAM is in a stretched state, and the hydrogel swells; when the temperature is higher than LCST, the polymer chains of PNIPAM tend to form hydrogen bonds by themselves, expel water, and the polymer network collapses [1,2]. Recently, a bilayer-structured actuator based on PNIPAM and polydimethylsiloxane (PDMS) has been developed to achieve multiple stimuli responsiveness to light, electricity, temperature, water, ions, and organic solvents. The device also enables programmable complex deformation under multiple simultaneous or sequential stimuli [28]. Spiropyran molecules are a class of photochromic organic compounds that are widely studied. The energetically stable spiro (SP) form changes to the zwitterionic merocyanine (MC) form as a result of UV light irradiation, causing a dramatic change in the hydrophilic–hydrophobic nature accordingly [29,30]. The swelling properties of the two monolayer hydrogels under the stimuli of heat, UV light, acid, and metal ions can be different, resulting in different bending behaviors. In particular, by introducing a second network into the hydrogel macromolecular network, the energy dissipation in the network can be effectively improved, thereby the mechanical properties of the hydrogel can be enhanced [31]. Scheme 1 illustrates our new strategy and the fabrication process of the bilayer hydrogel. Such multistimuli-responsive high-strength bilayer hydrogels are expected to provide valuable avenues in soft actuators and smart switches.

## 2. Materials and Methods

### 2.1. Materials

Photochromic acrylate derivatives of functional spiropyran groups (SPMA) were obtained from our previously published literature [14]. 2-Acrylamide-2-methyl propane sulfonic acid (AMPS, 98%), α-ketoglutaric acid (KA, BR), *N, N′*-methylene bis-(acrylamide) (MBAA, 99%), Tween 80 (CP), and sodium hydroxide (NaOH, AR) were supplied by Sinopharm Chemical Reagent Co., Ltd. Methyl acrylate (MA, 98.5%) and *N*-isopropylacrylamide (NIPAM, 98%) were purchased from Macklin Bio-Chem Technology Co., Ltd. (Shanghai, China). Phenyl bis(2,4,6-trimethylbenzoyl)-phosphine oxide (PBPO, 97%) was purchased from Aladdin Bio-Chem Technology Co., Ltd. (Shanghai, China). NIPAM was purified by recrystallization from hexanes. Deionized water (18.2 Ω-cm resistivity at 25 °C) was used in all experiments.

### 2.2. Preparation of Monolayer Hydrogels

The preparation of PNaAMPS powders occurred as follows: AMPS (4.145 g, 1 M), MBAA (0.1233 g, 0.04 M), NaOH (0.9 g), and KA (0.0029 g, 0.001 M) were added to a three-neck flask containing deionized (DI) water of 20 mL, and a transparent precursor solution was obtained after the mixture was stirred and dissolved. The solution was poured into a mold composed of a pair of glass sheets and silicone gaskets (thickness of 1 mm), where polymerization occurred under ultraviolet (UV) light radiation for 7 h, producing poly(2-acrylamide-2-methyl propane sulfonate acid sodium) (PNaAMPS) hydrogel. After drying in an oven to a constant weight, the PNaAMPS xerogel was ground into powders with particle sizes of fewer than 200 μm through an agate mortar.

The preparation process of P(NIPAM-*co*-MA-*co*-SPMA) hydrogel for control occurred as follows: an aqueous phase consisted of NIPAM (1.8106 g, 2 M), Tween 80 (0.08 g, 1 wt% of DI water) and MBAA (0.0025 g, 0.002 M) in 8 mL of DI water, which were uniformly mixed until the solution was clear and transparent. An oil phase consisted of SPMA (0.0063 g, 0.002 M), MA (0.181 g, 10 wt% of NIPAM) and PBPO (0.0201 g, 0.006 M), which were ultrasonically mixed until the solution was transparent. Then, the oil phase was added dropwise to the aqueous phase (1:43, in volume) for further emulsification. After the precursor solution was uniformly emulsified, the precursor solution was injected into a reaction cell (100 mm × 100 mm) composed of a pair of glass sheets and silicon gaskets spacer with a thickness of 1 mm. The P(NIPAM-*co*-MA-*co*-SPMA) hydrogel was obtained by white light irradiation at 5 °C for 6 h.

The preparation process of PNaAMPS/P(NIPAM-*co*-MA-*co*-SPMA) hydrogels for control follows the similar procedures; in this case, PNaAMPS powders (0.12 g, 0.015 g/mL) were added into the aqueous phase to introduce the second layer network. In addition, PNaAMPS/P(NIPAM-*co*-MA) hydrogel without SPMA monomer was also prepared.

### 2.3. Preparation of the Bilayer Hydrogel

Firstly, the bottom monolayer hydrogel sheet was prepared. A precursor solution consisted of PNaAMPS powders; NIPAM, MA, MBAA, and KA with the same amounts as above were poured into a self-made glass mold isolated by silicone gasket (thickness of 1 mm). Then, the precursor solution was irradiated under white light at 5 °C for 6 h to obtain a PNaAMPS/P(NIPAM-*co*-MA) hydrogel sheet. Afterwards, the covering glass slide was lifted and another thin silicone gasket was placed between the bottom gel sheet and covering glass slide. Finally, a precursor solution consisted of PNaAMPS powders; NIPAM, MA, MBAA, SPMA, and KA with the same amounts as above were injected onto the bottom gel sheet. The bilayer hydrogel named PNaAMPS/P(NIPAM-*co*-MA-*co*-SPMA)-PNaAMPS/P(NIPAM-*co*-MA) was obtained after the precursor solution was irradiated under white light for additional 6 h. After polymerization, the obtained bilayer hydrogel was washed by deionized water thoroughly.

### 2.4. Tensile Measurement

Uniaxial tensile tests were carried out with rectangular strips (30 mm × 5 mm) using a universal tensile testing machine (CMT6103, MTS Co. Ltd., Shanghai, China) equipped with a 1 kN load cell at a constant stretching velocity of 100 mm min^−1^ at room temperature. Elastic modulus *E* was calculated from the slope of the initial linear region of the stress–strain curve (within 5~10%). Work of extension was calculated from the area under the stress–strain curves up to fracture of an uncut sample. The nominal stress *σ* was calculated from the tensile force and the initial cross-section area of the undeformed sample. The strain rate *ε* was defined as the ratio of stretching velocity to the original gauge length. Three measurements were performed for each sample unless mentioned otherwise.

### 2.5. Equilibrium Swelling Ratio of the Hydrogels

The swelling properties of the hydrogels were measured over a range of temperatures. Firstly, the hydrogel samples were placed into DI water and maintained under different temperatures to fully swell. Then, the swollen hydrogels were taken out of DI water and the surface was wiped by a filter paper to measure the sample weight (*W*_s_). Finally, the sample was thoroughly dried in an oven at 100 °C to measure the sample weight (*W*_d_). Equilibrium swelling ratio (*ESR*) was determined as

(1) $$\mathrm{ESR}\;(\mathrm{wt}\%)=\frac{W_{s}\;{-\;W}_{d}}{W_{d}}\;\times \;100\%.$$

### 2.6. Relative Swelling Test of the Hydrogels

The relative swelling (*RS*) is the change in the volume of hydrogel with time after external stimulation. The *V*_t_ is the volume of the hydrogel sample after being immersed in water for a period of time; *V*_0_ is the volume of the hydrogel sample after swelling and equilibrium in water.

(2) $$\mathrm{RS}\;(\mathrm{wt}\%)=\frac{V_{t}\;{–\;V}_{0}}{V_{0}}\;\times \;100\%.$$

### 2.7. Bending Degree of the Hydrogels

The bending process of a bilayer hydrogel strip (30 mm × 2 mm × 2 mm) soaking in solvents was recorded using a cell phone. The bending degree of the bilayer hydrogel was measured on the images using Image J software. As shown in Figure S1, take a cross section as the benchmark and establish a coordinate system to connect the interface at both ends of the bilayer hydrogel. The angle obtained is the bending angle of the bilayer hydrogel. In this work, the bending toward the PNaAMPS/P(NIPAM-*co*-MA-*co*-SPMA) upper layer is designated a positive bending degree, and a negative bending degree represents the direction of the bending toward the PNaAMPS/P(NIPAM-*co*-MA) bottom layer. The bending and recovery experiment were repeated several times to determine the cycle performance.

### 2.8. Microscopic Fourier Transform Infrared (Micro-FTIR) Spectroscopy

The bilayer hydrogel was placed in a freeze dryer for 12 h to dehydrate the sample to xerogel. Scanning was performed on a micro-FTIR spectrometer (Thermo Fisher Scientific/Nicolet iN10, Waltham, MA, USA) for spectral analysis. In order to record the distribution of nitro group inner spiropyran component at the interfaces between the two hydrogel sheets, the absorption peak of nitro symmetric stretching vibration at 1350 cm^−1^ was collected.

### 2.9. Ultraviolet Test

Photoisomerization of the hydrogel was characterized by a UV–vis spectrophotometer (Hitachi U-3900). All samples were treated with UV light by irradiating photochromic hydrogels with a UV curable machine (a mercury lamp with a maximum at 365 nm and an intensity of 5.3 mW cm^−2^, ELC-500, Electro-Lite Co., Bethel, CT, USA). The UV–vis spectra of the samples were initially collected following visible light irradiation (white light LED lamp (OPPLE, 55 W, with an intensity of 0.8 mW cm^−2^)). Then, the sample was irradiated by UV light for different time intervals and the corresponding UV–vis spectra of the samples were collected.

### 2.10. Scanning Electron Microscopy (SEM)

The microstructure of the bilayer hydrogel was observed by Hitachi SU8010 (Hitachi Limited Co, Hitachi, Japan) SEM. In detail, the sample was frozen and fractured in liquid nitrogen and then freeze-dried for 36 h. Afterwards, the fractured surface of the sample was coated with a thin layer of gold by the sputtering method before SEM characterization. The accelerating voltage for SEM was 5.0 kV.

## 3. Results and Discussion

### 3.1. Mechanical Properties of Hydrogels

**Figure 1:**
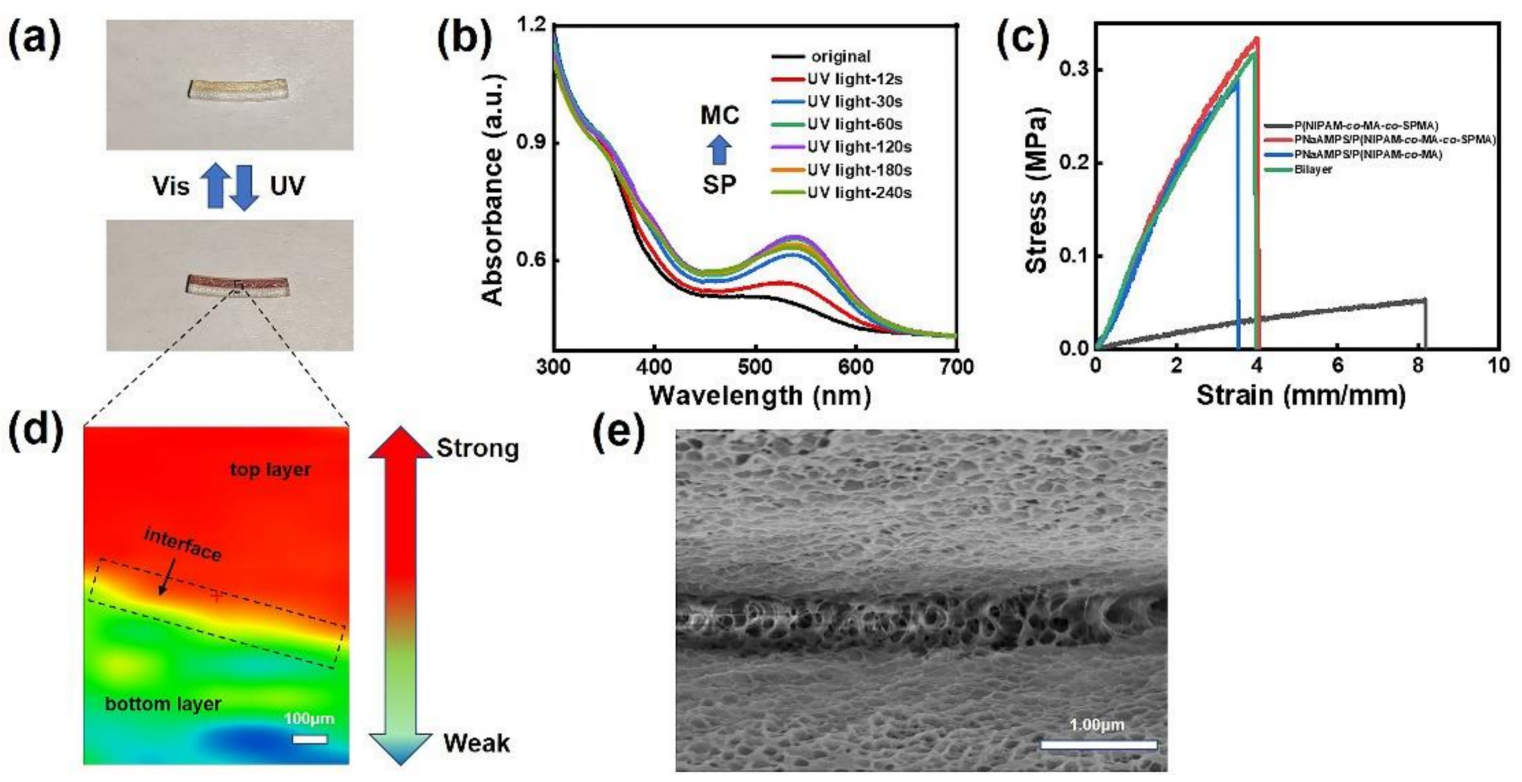
(**a**) Comparison of the bilayer hydrogel before and after UV light irradiation; (**b**) coloring kinetics of PNaAMPS/P(NIPAM-*co*-MA-*co*-SPMA) hydrogel; (**c**) tensile stress–strain curves of four hydrogels; (**d**) the micro-FTIR distribution map of the specified area scanned at 1350 cm^−1^; (**e**) SEM cross-sectional images of bilayer hydrogel structure (The top layer is PNaAMPS/P(NIPAM-*co*-MA-*co*-SPMA) hydrogel and the bottom layer is PNaAMPS/P(NIPAM-*co*-MA) hydrogel).

The following hydrogels were prepared: P(NIPAM-*co*-MA-*co*-SPMA), PNaAMPS/P(NIPAM-*co*-MA), PNaAMPS/P(NIPAM-*co*-MA-*co*-SPMA) hydrogels and PNaAMPS/P(NIPAM-*co*-MA-*co*-SPMA)-PNaAMPS/P(NIPAM-*co*-MA) bilayer hydrogels. As shown in Figure 1a, after UV irradiation, an obvious bilayer structure was observed: the PNaAMPS/P(NIPAM-*co*-MA-*co*-SPMA) top layer appeared purple on account of conversion of spiropyran from spiro (SP) form to zwitterionic merocyanine (MC) form [14], meanwhile the PNaAMPS/P(NIPAM-*co*-MA) bottom layer was slightly transparent white. The coloring process of the top PNaAMPS/P(NIPAM-*co*-MA-*co*-SPMA) sheet at different irradiation time was shown in Figure 1b. The two layer sheets were well bonded at the interfaces and cannot be separated even after repeat bending. This assumption was proved by microscopic Fourier transform infrared microscopy (Figure 1d), where a clear color fusion that overlapped red with green luminescence appeared. Tensile tests were carried out on these hydrogels by a universal tensile testing machine, and the test results are shown in Figure 1c and Table 1. According to the stress–strain curves, the failure stress, failure strain, and tensile work of the P(NIPAM-*co*-MA-*co*-SPMA) hydrogel reaches 54 kPa, 817% and 250 kJ m^−3^, respectively. Moreover, when a brittle/ductile dual network PNaAMPS was introduced, the mechanical properties of hydrogels were significantly improved. Specifically, the failure stress, tensile work and elastic modulus of PNaAMPS/P(NIPAM-*co*-MA-*co*-SPMA) were increased to 335 kPa, 752 kJ m^−3^ and 82 kPa, respectively. These remarkable enhancement in mechanical property are caused by the synergy effects between the asymmetric PNaAMPS and P(NIPAM-*co*-MA-*co*-SPMA) networks. This synergy effect can better resist the deformation of external forces and effectively dissipate energy. During deformation, the failure behaviors can be described in the following way: under small deformation, though the brittle PNaAMPS network was destroyed first, the broken PNaAMPS fragments could act as a physical cross-linking agent and attach to the soft and tough P(NIPAM-*co*-MA-*co*-SPMA) copolymer. In the network, large deformations were obtained by slippage of molecular chains. When the deformation was large enough, the physical cross-linking points with PNaAMPS fragments were further destroyed, and the P(NIPAM-*co*-MA-*co*-SPMA) copolymer network was gradually destroyed until the macroscopic failure. The mechanical properties of the hydrogels were mainly enhanced by dissipating energy through the brittle PNaAMPS network as “sacrificial bonds” [31].

Moreover, in Figure 1c, it can be observed that the failure stress of the bilayer hydrogel was between that of the two monolayer hydrogels, PNaAMPS/P(NIPAM-*co*-MA-*co*-SPMA) and PNaAMPS/P(NIPAM-*co*-MA), while the modulus of the bilayer hydrogel was slightly larger, indicating the synergistic effect and good integration of the bilayer hydrogel. Here, it needs to be mentioned that during stretching, the weaker hydrogel portion with a shorter elongation at break would fracture before the overall breaking of the integrated bilayer hydrogel [32]. However, the two layers were still nicely integrated during the entire tensile test without any observed separation at the interface. To explore the underlying mechanism behind this phenomenon, microscopic analysis of the bilayer hydrogel interface was performed under micro-FTIR spectroscopy. It can be seen from Figure 1d that in the absorption profile of the absorption peak of nitro symmetric stretching vibration at 1350 cm^−1^, the distribution of nitro group inner spiropyran component gradually decreased from top to bottom, and the existence of nitro group permeated at the interface of bilayer structure, while only the top hydrogel contained the nitro group, thus demonstrating the influence of this permeation effect on the mechanical properties of bilayer hydrogels. Furthermore, Figure 1e shows the microstructure at the interface of the bilayer hydrogel. It can be observed that there is a typical porous microstructure with a thickness of approximately 0.5 μm at the interface between the two layers of the bilayer hydrogel, which tightly joins the two layers. The interfacial adhesion between bilayer hydrogel is strong due to the covalent bonds formed. In all the tests, no delamination has been observed. If there is no such microstructure, it is easy to observe an obvious cracked gap at the interface, and delamination occurs after two bending cycles [21].

### 3.2. Thermo-Responsive Bending Behavior

**Figure 2:**
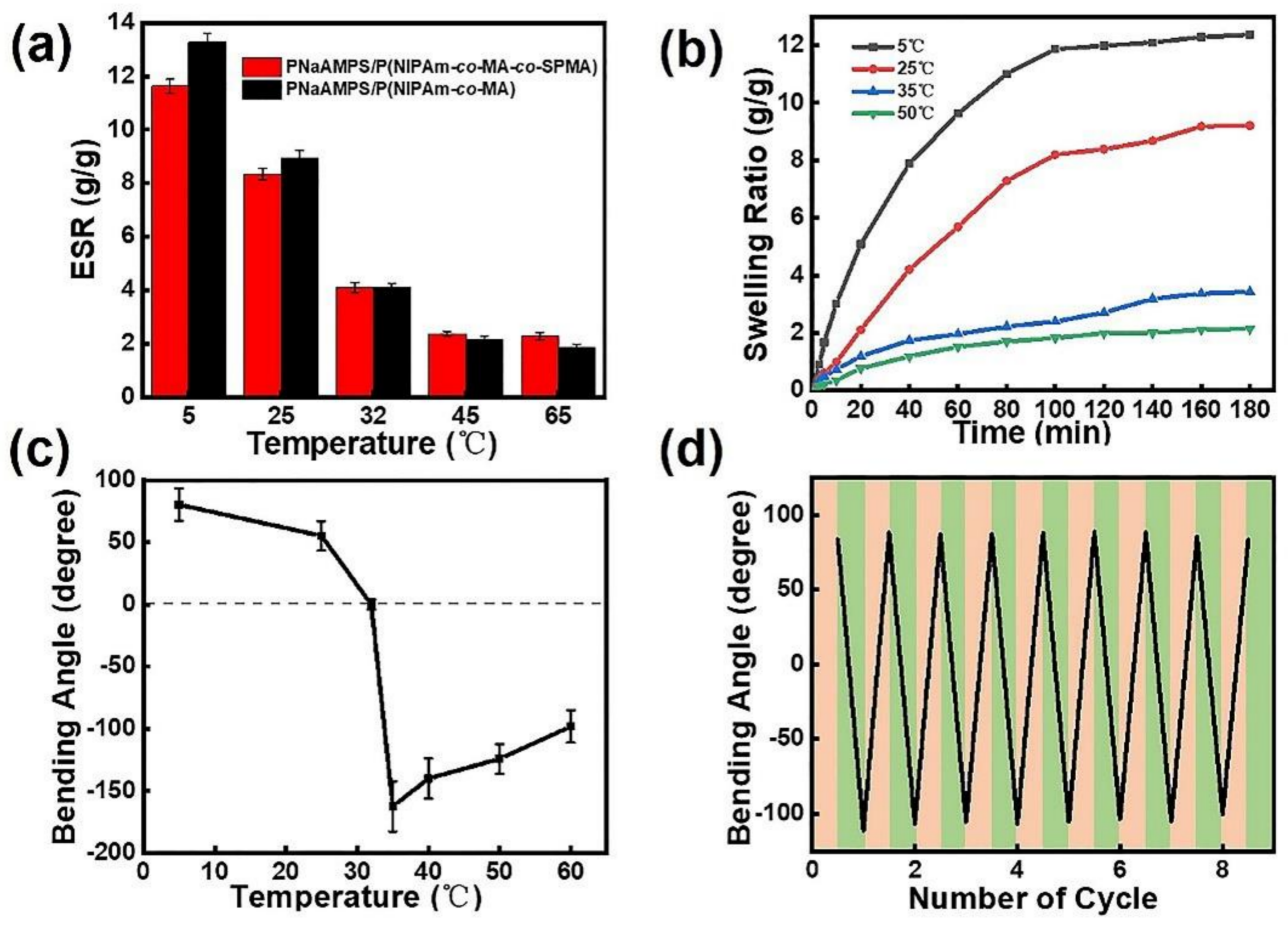
(**a**) ESR of hydrogels in DI water at different temperatures; (**b**) swelling curve of PNaAMPS/P(NIPAM-*co*-MA-*co*-SPMA) hydrogel in DI water at different temperatures; (**c**) final bending angles of bilayer hydrogels in DI water at different temperatures; (**d**) cyclic reversible shape-changing behavior of bilayer hydrogel upon the temperature changing between 5 and 60 °C (Green represents the transfer of hydrogel from 5 °C to 60 °C, pink represents the transfer of hydrogel from 60 °C to 5 °C).

Both layers of the bilayer hydrogel consist of NIPAM with LCST phase separation; therefore, the two layers would undergo similar thermo-responsive behaviors with temperature. We first characterized the equilibrium swelling ratio of the PNaAMPS/P(NIPAM-*co*-MA-*co*-SPMA) hydrogel and the PNaAMPS/P(NIPAM-*co*-MA) hydrogel in various temperatures. From Figure 2a, it can be seen that hydrogels have higher ESR at low temperature, while ESR drops sharply at high temperature. When the temperature is lower than 32 °C, the hydrated H-bond formed by amide group and water in the hydrogel network is dominant, the molecular chain extends. When it is higher than 32 °C, the hydrophobic interaction of isopropyl group is dominant, and the amide group tends to form intramolecular or intermolecular H-bonds with itself, which intensifies the hydrogel network. NIPAM chains underwent a conformational change from an extended hydrated coil to a collapsed hydrophobic globule. In addition, after comparing the ESR of PNaAMPS/P(NIPAM-*co*-MA-*co*-SPMA) hydrogel and PNaAMPS/P(NIPAM-*co*-MA) hydrogel, we determined that the ESR of PNaAMPS/P(NIPAM-*co*-MA-*co*-SPMA) hydrogel is slightly lower than that of the PNaAMPS/P(NIPAM-*co*-MA) hydrogel when it is lower than 32 °C, but when the temperature is higher than 32 °C, the result is opposite. This is mainly due to the hydrophobicity of the spiro (SP) form of spiropyran; the LCST can be reduced by the incorporation of hydrophobic groups [29]. At the same time, the reduced temperature for phase transition may be attributed to gradual thermal precipitation by the PNaAMPS/P(NIPAM-*co*-MA-*co*-SPMA) copolymer with hydrophobic spiropyran. The swelling curve of PNaAMPS/P(NIPAM-*co*-MA-*co*-SPMA) hydrogel in DI water at different temperatures is presented in Figure 2b, showing similar results.

It is well known that the shape transformation behaviors of hydrogel actuators are essentially determined by their anisotropic structures. To investigate the response performance of the thermo-responsive bilayer hydrogel, we further characterized the swelling-induced bending behavior of bilayer hydrogel strip by measuring the bending angle. For comparison, the bending angle of the bilayer hydrogel strip to the PNaAMPS/P(NIPAM-*co*-MA-*co*-SPMA) layer was marked as a positive value. The numerical value of the final bending angle of the bilayer hydrogel in DI water at different temperatures is evaluated in Figure 2c. As shown in Figure 2a, the ESR of PNaAMPS/P(NIPAM-*co*-MA-*co*-SPMA) hydrogel is lower than PNaAMPS/P(NIPAM-*co*-MA) hydrogel at 5 °C, so the bilayer hydrogel is bending towards the PNaAMPS/P(NIPAM-*co*-MA-*co*-SPMA) layer spontaneously with a bending angle of 83.4°. With increasing temperature, the disparity of swelling ratio between two layers decreases. This leads to the decreasing bending angle of the bilayer, and the whole structure becomes almost flat at 32 °C. Due to the change in trend of swelling ratio between two layers with further increasing temperature, the bilayer then bends in the opposite direction with a bending angle of as much as −162.4° at approximately 35 °C. The bending angle value gradually decreases when the temperature is higher than 35 °C. It is plausibly due to that the increase in temperature and the deepening of the phase separation of PNIPAM restricted the deformation behavior of the bilayer hydrogel. Generally, upon stimuli, the differences in swelling/shrinking between the two layers will cause the swelling/shrinking of one layer to be constrained by the other layer [2]. Therefore, the difference in ESR between the two layers of hydrogel leads to the deepening of phase separation of PNIPAM during thermo-response, which affects the internal stresses of the bilayer hydrogel, thus limiting its deformation. Figure S2 shows the photographs of the corresponding hydrogel at different temperatures.

Next, we examined the recyclability of the bilayer hydrogel upon the temperature changing between 5 °C and 60 °C. Figure 2d shows that the bilayer hydrogel exhibits good repeatable responsiveness at two temperatures, 5 °C and 60 °C, and the responsiveness remains good after multiple cycles. The above results clearly demonstrate our success in the fabrication of thermo-responsive bilayer hydrogel actuators that show tunable deformation extent and excellent recyclability. In addition to the programmable deformations of strips, the bilayer hydrogel could also be programmed to achieve a complex shape deformation (Figure S3).

### 3.3. UV Light and Acid Responsive Bending Behavior

Spiropyrans are interesting photoswitches that interconvert between two isomers, a ring-closed spiropyran form (SP) and ring-opened merocyanine form (MC), which differ in dipole moment, hydrophobicity and net charge. Recent work has shown that the incorporation of spiropyrans in materials can cause significant changes in their physical and chemical properties upon irradiation [33]. Figure 1b shows the coloring kinetics of PNaAMPS/P(NIPAM-*co*-MA-*co*-SPMA) hydrogel. From the coloring kinetics of hydrogel, it was determined that the SP ring opening characteristic peak gradually increased with the extension of UV irradiation time at 500 nm~600 nm, indicating that SP isomer gradually transformed into MC isomer, and MC concentration was the largest at 120 s, showing a good photochromic behavior. As shown in Figure S4, the SP ring opening characteristic peak gradually weakens with the extension of visible light irradiation time in the fading kinetics of PNaAMPS/P(NIPAM-*co*-MA-*co*-SPMA) hydrogel, indicating that the MC isomer is transformed into SP isomer again, and the results show that the hydrogel has an excellent reversible photo-responsive behavior.

**Figure 3:**
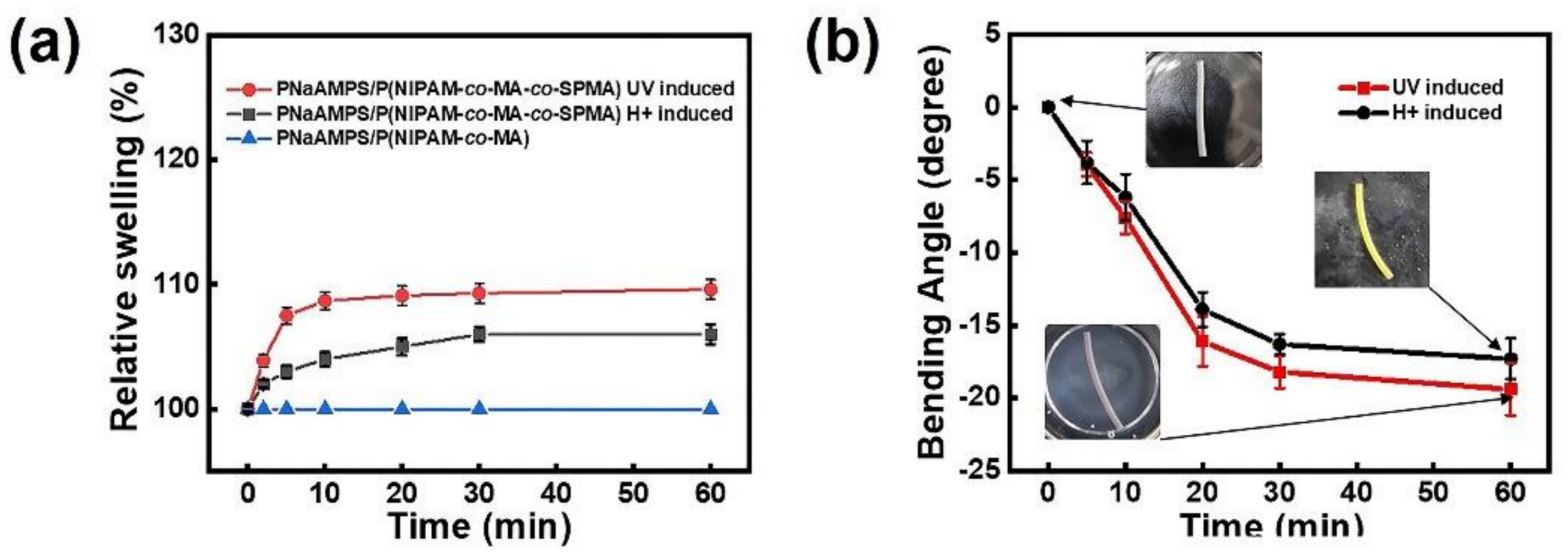
(**a**) The curve of relative swelling ratio of PNaAMPS/P(NIPAM-*co*-MA-*co*-SPMA) hydrogel and PNaAMPS/P(NIPAM-*co*-MA) hydrogel at 32 °C in DI water under UV light and H+ stimulation (0.05 M) with time; (**b**) UV light and H+ (0.05 M) responsive bending angle at 32 °C in DI water.

The SPMA monomers in the PNaAMPS/P(NIPAM-*co*-MA-*co*-SPMA) hydrogel network can change the structure by UV light irradiation or H+, specifically from the hydrophobic SP isomer to the hydrophilic MC isomer, yielding the resulting polymers’ switch hydrophobicity and hydrophilicity. First, Figure 3a shows the relative swelling kinetics of the hydrogels under UV light or H+ (0.05 M) stimulation. The test process is as follows: the hydrogel sample was first swelled at 32 °C in DI water to equilibrium and then exposed to UV light or H+ environment so as to ensure the SP isomer in the network was converted into the MC isomer and increased the hydrophilicity of the sample; then, the relative swelling ratio versus time was recorded. The “time” here represents the total time obtained by placing the hydrogel in a dark environment for 2 min after the first UV irradiation, and then continuing the UV irradiation for 2 min. UV irradiation is carried out at intervals to ensure that there are a large number of hydrophilic MC isomers in the network. The hydrogels with swelling balance continued to swell under the stimulation of UV light or acid and the swelling process completed in 30 min with a good swelling ratio. What is more, because the PNaAMPS/P(NIPAM-*co*-MA) hydrogel does not contain spiropyran, its relative swelling ratio under UV light and H+ environment remains unchanged.

For the bilayer hydrogel, the PNaAMPS/P(NIPAM-*co*-MA-*co*-SPMA) layer tends to swell further under UV light or H+ environment while the volume of PNaAMPS/P(NIPAM-*co*-MA) layer remains almost unchanged. We observed that the mismatch in swelling properties of active and passive hydrogel in bilayers caused the bilayer hydrogel to bend toward PNaAMPS/P(NIPAM-*co*-MA) layer.

As shown in Figure 3b, when the bilayer hydrogel is exposed to UV light or H+ environment at 32 °C in DI water, the bending angle changes rapidly at approximately −15° in the first 30 min. This phenomenon is consistent with the swelling behavior of PNaAMPS/P(NIPAM-*co*-MA-*co*-SPMA) hydrogel in Figure 3a. Later on, the bending angle of the bilayer hydrogel gradually reaches −19.4° and −17.3°, respectively, under UV light and H+ environment in the following hour of observation. Generally, larger difference in hydrogels in response to stimulus could produce a stronger driving force to cause a larger deformation of the bilayer hydrogels, thus the bending angle of bilayer under UV light irradiation is slightly larger than that of H+ owing to the larger relative swelling ratio in Figure 3a. The increase in charge density in the system under UV irradiation is greater than that under H+ environment, resulting in greater deformation under UV irradiation [33]. Figure S5 shows cyclic actuating behaviors of the bilayer hydrogels under the stimulus of UV light changes at 32 °C in DI water, which are highly reproducible in the investigated eight cycles.

### 3.4. Metal Ion Responsive Bending Behavior

Owing to the reversible photo-cleavage of the spiro C-O bond of spiropyran, the colorless ring-closed spiropyran (SP) isomer could switch into colorful ring-opened merocyanine (MC) isomer which could be utilized for the visual detection of multiple metal ions [34].

**Figure 4:**
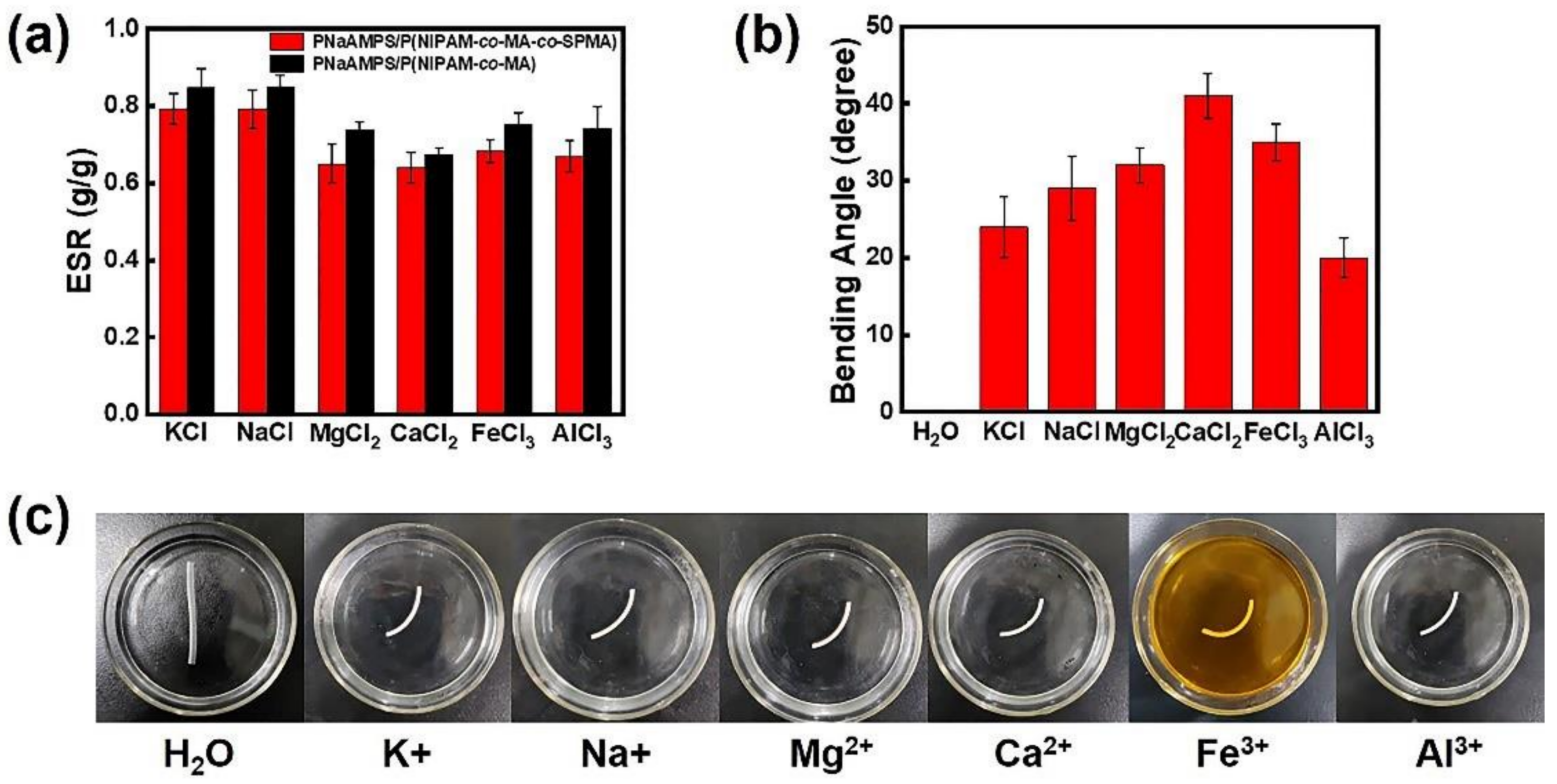
(**a**) ESR of monolayer hydrogels in different cation (K+, Na+, Mg^2+^, Ca^2+^, Fe^3+^, Al^3+^)-containing aqueous solutions of 0.5 M at 32 °C; (**b**) bending angles of bilayer hydrogels in different cation (K+, Na+, Mg^2+^, Ca^2+^, Fe^3+^, Al^3+^)-containing aqueous solutions of 0.5 M at 32 °C; (**c**) photographs of the corresponding bilayer hydrogels in different metal ion solutions of 0.5 M at 32 °C.

We characterized the ESR of two layers in different metal ion solutions in Figure 4a. To exclude the influence of other factors, the test was conducted at 32 °C. It can be observed that the ESR of PNaAMPS/P(NIPAM-*co*-MA-*co*-SPMA) hydrogel in different metal ion solutions is lower than that of PNaAMPS/P(NIPAM-*co*-MA) hydrogel, which is due to the combination of spiropyran and metal ions, which causes the hydrogel to discharge some water, thus reducing the ESR. What is more, it is noteworthy that the ESR values are much smaller than ESR values in Figure 2a. This is because the sulfonate group on PNaAMPS and the amide group of P(NIPAM-*co*-MA-*co*-SPMA) can crosslink with metal ions to form metal coordination interactions, so in the metal ion solution, the network structure of the hydrogel becomes more dense, and ESR decreases.

We then investigated the bending behavior of bilayer hydrogels at different solvent compositions. On one hand, compared with in DI water, bending angles are produced in different metal ion solutions, as shown in Figure 4c; the length of bilayer hydrogel undergoes a shrink with the addition of different cations in aqueous solutions. On the other hand, the fact that there is no obvious difference in the bending angle between monovalent and divalent metal ions is a peculiar phenomenon. However, when the valence state of metal ions increases to trivalent, the bending angle tends to decline (Figure 4b). With the increase in the valence state of metal ions, metal ions combine with more sulfonate and amide groups, which enlarges the crosslinking density of the hydrogel network and reduces the swelling ratio, thus reducing its bending angle. At the same time, spiropyran form complexes with higher valence metal ions, which also causes this result [35].

## 4. Conclusions

In summary, we have developed a convenient strategy to fabricate an anisotropic hydrogel actuator with multistimuli-responsive behaviors. The UV light irradiation/pH-responsive as well as thermo-responsive PNaAMPS/P(NIPAM-*co*-MA-*co*-SPMA) hydrogel and thermo-responsive shape-changing only PNaAMPS/P(NIPAM-*co*-MA) hydrogel can be well bonded to obtain planar sheet. Benefitting from the phase separation with LCST of PNIPAM and remarkable change in the hydrophilic–hydrophobic nature of spiropyran component, the bilayer hydrogel sheet could be facilely controlled by environmental stimuli such as temperature change, UV light irradiation, H+, and metal ions. Reversible shape deformations of the actuators were also demonstrated based on inhomogeneous swelling that resulted in diverse bending angles. In addition, the bilayer sheets of hydrogels were strengthened via double networks (DN). Our work will inspire the design and fabrication of novel soft actuators integrating more intelligent functions.

## Figures and Tables

**Table 1:** Comparison of mechanical properties for four kinds of hydrogels.

Sample	*σ* (kPa)	*ε* (%)	*E* (kPa)	*W* (kJ m^−3^)
P(NIPAM-*co*-MA-*co*-SPMA)	54 ± 9	817 ± 208	9 ± 4	250 ± 27
PNaAMPS/P(NIPAM-*co*-MA-*co*-SPMA)	335 ± 26	398 ± 35	82 ± 17	752 ± 49
PNaAMPS/P(NIPAM-*co*-MA)	289 ± 31	352 ± 51	69 ± 18	556 ± 30
Bilayer hydrogel	318 ± 22	395 ± 44	97 ± 21	692 ± 61

*σ, ε, E* and *W* refer to tensile strength, elongation at break, elastic modulus, and work of extension, respectively.

## Data Availability

Not applicable.

## Supplementary Materials

The following supporting information can be downloaded at: https://www.mdpi.com/article/10.3390/polym15030786/s1, Figure S1: The measurement of bending angle for the bilayer hydrogel; Figure S2: Photographs of the corresponding bilayer hydrogels in DI water at different temperatures; Figure S3: Flowerlike soft actuator based on the bilayer hydrogel; Figure S4: Fading kinetics of PNaAMPS/P(NIPAM-*co*-MA-*co*-SPMA) hydrogel; Figure S5: Cyclic reversible shape-changing behavior of bilayer hydrogel of the turning on/off of UV light at 32 °C in DI water (Green represents the turning off of UV light, pink represents the turning on of UV light).
