# Supplementary material for: Designing Multistimuli-Responsive Anisotropic Bilayer Hydrogel Actuators by Integrating LCST Phase Transition and Photochromic Isomerization
Source: Polymers (Basel). 2023 Feb 3;15(3):786. doi: 10.3390/polym15030786 (PMC9918905; doi:10.3390/polym15030786)
Supplement: Supplementary file 1 [file polymers-15-00786-s001.zip › polymers-2183954-supplementary.pdf]

Supporting Information

Designing Multistimuli-Responsive Anisotropic Bilayer Hydrogel Actuators by Integrating LCST Phase Transition and Photochromic Isomerization

Shijun Long [1,2], Jiacheng Huang [1,2], Jiaqiang Xiong [1,2], Chang Liu [1,2], Fan Chen [1,2], Jie Shen [3,*], Yiwan Huang [1,2,*] and Xuefeng Li [1,2,*]

1 Hubei Provincial Key Laboratory of Green Materials for Light Industry, Hubei University of Technology, Wuhan 430068, China
2 New Materials and Green Manufacturing Talent Introduction and Innovation Demonstration Base, Hubei University of Technology, Wuhan 430068, China
3 Hubei Research and Design Institute of Chemical Industry, Wuhan 430073, China
* Correspondence: j_shen21@163.com (J.S.); yiwanhuang@hbut.edu.cn (Y.H.); li_xf@mail.hbut.edu.cn (X.L.)

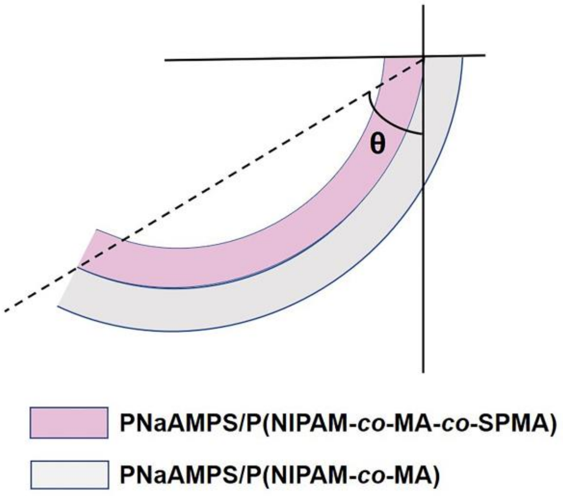

Figure S1. The measurement of bending angle for the bilayer hydrogel.

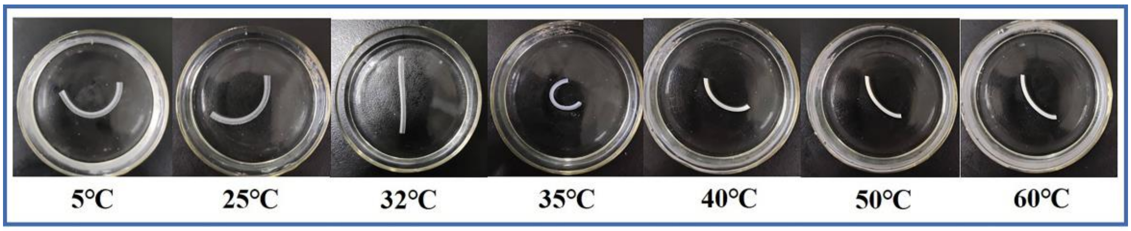

Figure S2. Photographs of the corresponding bilayer hydrogels in DI water at different temperatures.

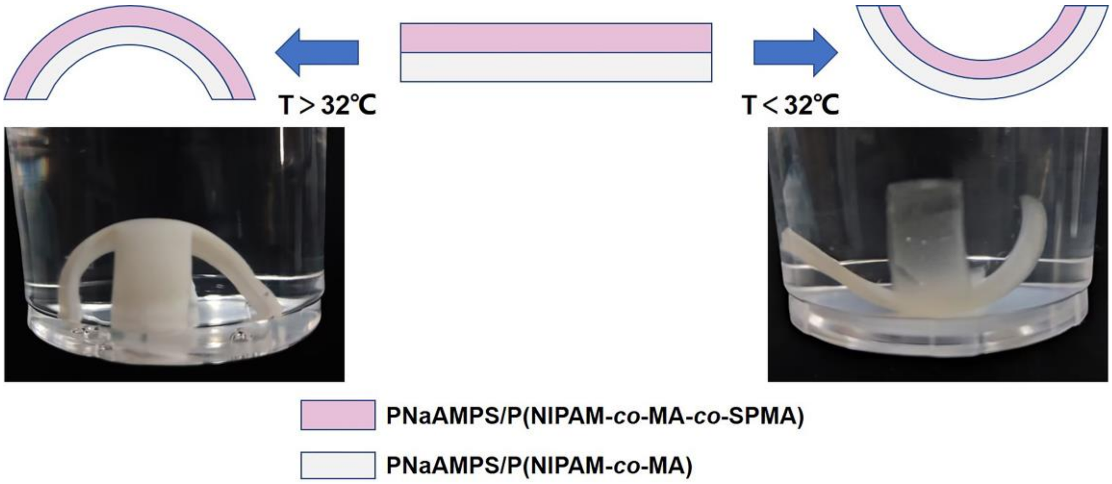

Figure S3. Flowerlike soft actuator based on the bilayer hydrogel.

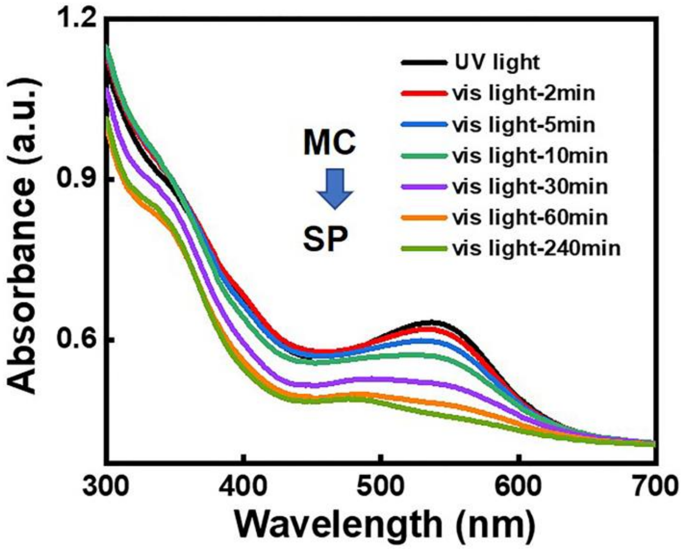

Figure S4. Fading kinetics of PNaAMPS/P(NIPAM-co-MA-co-SPMA) hydrogel.

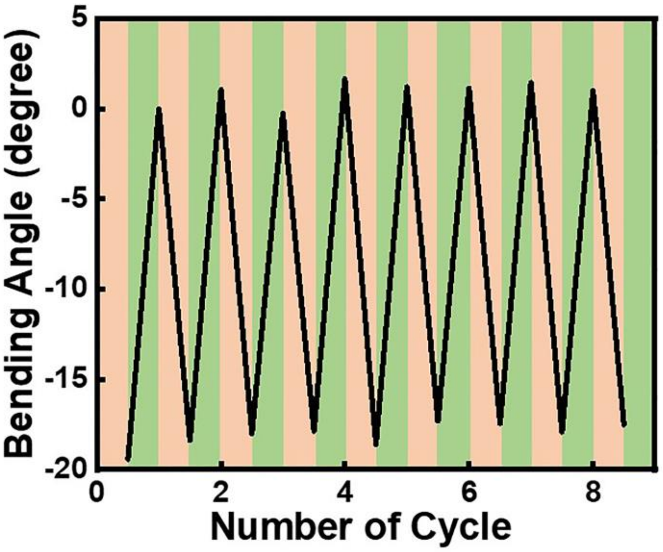

Figure S5. Cyclic reversible shape-changing behavior of bilayer hydrogel of the turning on/off of UV light at 32°C in DI water (Green represents the turning off of UV light, pink represents the turning on of UV light).
